# Comparative Assessment of Uterocervical Angle Using Transvaginal, Transabdominal, and Transperineal Ultrasonography Between 16 and 24 Weeks of Gestation

**DOI:** 10.3390/diagnostics15172146

**Published:** 2025-08-25

**Authors:** Emrah Dagdeviren, Can Tercan, Ali Selcuk Yeniocak, Busra Cigdem, Elif Ataseven, Akin Varlik, Mehmet Fatih Kilic, Yucel Kaya

**Affiliations:** 1 Department of Obstetrics and Gynecology, Basaksehir Cam and Sakura City Hospital, Istanbul 34480, Turkey; cntrcn89@gmail.com (C.T.); a.s.yeniocak@hotmail.com (A.S.Y.); drbusracigdem@gmail.com (B.C.); dr.elifataseven@gmail.com (E.A.); akinvarlik@yahoo.com (A.V.); 2 Department of Obstetrics and Gynecology, Merkezefendi State Hospital, Manisa 45120, Turkey; drmfatihkilic@gmail.com; 3 Division of Perinatology, Department of Obstetrics and Gynecology, Basaksehir Cam and Sakura City Hospital, Istanbul 34480, Turkey; yucelkaya0007@gmail.com

**Keywords:** agreement, consistency, correlation, transabdominal ultrasound, transperineal ultrasound, transvaginal ultrasound, uterocervical angle

## Abstract

**Objectives:** To assess the correlation and agreement of uterocervical angle (UCA) measurements obtained via transabdominal (TAUS), transperineal (TPUS), and transvaginal ultrasound (TVUS) between 16 and 24 weeks of gestation. **Methods:** In this prospective cross-sectional study, 136 pregnant women underwent UCA and cervical length (CL) assessments using TVUS, TAUS, and TPUS. All measurements were performed by a single experienced operator with an empty bladder. Correlation was assessed using Pearson analysis, while consistency and agreement were evaluated with intraclass correlation coefficient (ICC) and Bland–Altman plots. **Results:** Mean UCA values differed significantly between modalities (*p* < 0.001). Moderate correlation (r = 0.547) and consistency (ICC = 0.545) were found between TAUS and TVUS. The Bland–Altman analysis showed a systematic bias of –8° between TAUS and TVUS, with wide limits of agreement (–64° to +32.6°). TPUS showed a higher correlation with TVUS (r = 0.686), but poor consistency (ICC = 0.052), with broader limits of agreement (–32° to +49.2°). **Conclusions:** Although both TAUS and TPUS showed significant correlation with TVUS for UCA measurement, only TAUS demonstrated moderate consistency, suggesting that it should be used cautiously in clinical practice. TPUS demonstrated considerable variability in individual assessments and does not seem appropriate as a replacement for TVUS. Further studies are needed to validate these findings and improve measurement reliability.

## 1. Introduction

Preterm birth refers to all births occurring before 37 completed weeks of gestation or fewer than 259 days from the first day of the last menstrual period [1]. Approximately 10% of all births are preterm deliveries [2]. Two-thirds of infant deaths occur in preterm infants, and complications related to prematurity account for approximately one-third of all infant mortality [3]. The risk of complications such as severe intraventricular hemorrhage (grade 3–4), severe bronchopulmonary dysplasia, severe retinopathy of prematurity, and severe necrotizing enterocolitis increases as gestational age at the time of delivery decreases [4]. Preterm birth may occur spontaneously or may be indicated due to concerns regarding maternal or fetal conditions. Given that the etiology and underlying mechanisms vary among different phenotypes of preterm birth, this distinction should be acknowledged [5]. In selected patient groups, cerclage [6,7], and progesterone [8] have been recommended for reducing the risk of preterm birth and tocolysis [6] for delaying acute preterm birth. Therefore, identifying patients at high risk is essential for reducing perinatal morbidity and mortality.

Transvaginal ultrasonographic (TVUS) measurement of cervical length (CL) between 16 and 24 weeks of gestation is currently recommended for assessing spontaneous preterm birth risk [9,10]. In recent years, beyond CL, the clinical effects of anatomical angulations between the uterine corpus, cervix, and vagina have been investigated [11,12,13,14]. Among these, the uterocervical angle (UCA), also measured via TVUS, has emerged as a novel parameter for predicting spontaneous preterm birth, with wider angles associated with increased risk [11,12,15,16,17]. UCA refers to the angle between the cervical canal and the anterior uterine wall, and wide-angle values physiologically create a linear passageway. UCA may also be used together with CL and immune-mediated factors to improve the detection and prediction of spontaneous preterm birth. The UCA/CL ratio has likewise been proposed as a potentially predictive metric [18]. UCA has additionally been investigated as a predictor of labor induction success, suggesting broader clinical utility [19,20].

Although TVUS is considered the gold standard for CL measurement [10], it may be refused due to patient discomfort or embarrassment [21]. In contrast, transabdominal ultrasound (TAUS) offers ease of use and greater patient acceptability [10]. Several studies have demonstrated a positive correlation between TAUS and TVUS in CL measurement [22,23]. Similarly, transperineal ultrasound (TPUS) has been proposed as a viable alternative when TVUS is not feasible, either due to patient preference or limited resources [24]. TPUS has been used in some studies to assess uterine angulations [25,26]. Studies have also shown significant correlation between TPUS and TVUS for CL measurement [27,28].

While one study has investigated the correlation of UCA values obtained via TAUS and TVUS in pregnancies between 16 and 24 weeks of gestation, to date, no study has evaluated UCA measurement using TPUS [29].

### Objectives

The aim of this study is to evaluate the correlation and agreement of UCA measurements obtained by TAUS, TPUS, and TVUS in pregnant women between 16 and 24 weeks of gestation.

## 2. Materials and Methods

### 2.1. Study Design

This prospective cross-sectional study was conducted between September 2024 and June 2025. The study was conducted at a tertiary referral hospital providing specialized obstetric care. This study is a prospective, observational, and non-interventional cohort analysis that did not involve any therapeutic intervention; therefore, it was not registered in a clinical trial database, in accordance with the journal’s policy. The study was approved by the local institutional ethics committee (Approval No: KAEK/24/07/2024.75) and conducted in accordance with the ethical standards laid down in the Declaration of Helsinki. Written informed consent was obtained from all participants prior to their enrollment.

### 2.2. Participants

The study included pregnant women aged 18–40 years with singleton gestations and a gestational age between 16 + 0 and 23 + 6 weeks. Exclusion criteria included multiple gestation, fetal anomalies, established indications for cesarean delivery, history of spontaneous preterm birth, use of progesterone therapy, abnormal Pap smear results, placenta previa, maternal comorbidities such as diabetes or hypertension, prior cervical procedures (including LEEP, conization, cerclage, or dilation and curettage), uterine contractions, or a Bishop score greater than 6.

### 2.3. Variables

The primary variables assessed were CL and UCA, each measured via three ultrasound modalities: TVUS, TAUS, and TPUS.

### 2.4. Data Sources/Measurement

All ultrasound measurements were obtained after participants had emptied their bladders. Bladder emptying was standardized and ultrasonographically confirmed in all patients. TVUS and TPUS were performed in the dorsal lithotomy position, while TAUS was performed in the supine position. All ultrasonographic measurements were conducted by a single expert operator with 10 years of experience.

CL was measured as the distance between the internal and external os, following the technique outlined by the International Society of Ultrasound in Obstetrics and Gynecology (ISUOG) [30,31]. In cases of cervical curvature, the CL was calculated by summing the lengths of two or more segments [32]. Three measurements were taken, and the shortest reliable value was recorded as recommended [31].

**Figure 1:**
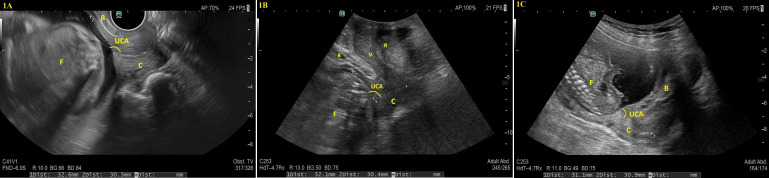
Representative ultrasound images of uterocervical angle (UCA) measurements using three different approaches. The UCA was defined as the angle between two lines: the first tracing the endocervical canal from internal to external os (drawn as a straight line even in curved canals), and the second extending from the internal os along the anterior uterine segment, ideally for 3 cm. (1**A**) demonstrates a transvaginal ultrasound taken at 21 weeks of gestation, (1**B**) shows a transperineal ultrasound at 20 weeks, and (1**C**) presents a transabdominal ultrasound at 19 weeks of gestation. The anatomical structures labeled in the images are as follows: B: Bladder, C: Cervix, F: Fetus, R: Rectum, UCA: Uterocervical Angle, V: Vagina.

UCA was measured from the same ultrasound frame used to assess CL; according to the method described by Dziadosz et al. [11], the UCA was defined as the angle between two lines: the first tracing the endocervical canal from internal to external os (drawn as a straight line even in curved canals) and the second extending from the internal os along the anterior uterine segment, ideally for 3 cm, in Figure 1. In cases of cervical funneling, the first line corresponded to the residual canal, while the second was drawn from the deepest funnel point to the anterior uterine wall.

For TVUS, a 4–9 MHz endocavitary vaginal probe was used. For TAUS and TPUS, a 1–5 MHz convex abdominal probe (C253) was employed using the Hitachi Arietta 65 ultrasound system (Tokyo, Japan). TAUS was performed with the participant in the supine position and the probe placed sagittally. TPUS was conducted either over the labia or with the labia gently separated; the probe was enclosed in a glove for hygiene and clarity [26]. Measurements were taken once the bladder, uterus, cervix, vagina, and rectum were adequately visualized on TPUS, following the protocol described by Dietz et al. [33].

### 2.5. Statistical Methods

Statistical analyses were performed using SPSS software (version 26.0.1; SPSS Inc., Chicago, IL, USA). The distribution of continuous variables was assessed using the Kolmogorov–Smirnov test. Data with a normal distribution were reported as mean ± standard deviation, compared using the ANOVA (Repeated Measures) test, and post hoc analysis was performed with the Bonferroni test. Non-normally distributed data were presented as medians (minimum–maximum), analyzed with the Friedman test, and post hoc analysis was performed using the Bonferroni-corrected Wilcoxon test. Categorical variables were expressed as numbers and percentages, and comparisons were made using the chi-square test. Pearson correlation analysis was used to assess the linear relationship between normally distributed continuous variables. The intraclass correlation coefficient (ICC) was calculated to assess the consistency and reliability between measurement methods. Agreement between measurement methods was further evaluated using non-parametric Bland–Altman plots, which illustrated the median difference (systematic bias) and the 2.5th and 97.5th percentiles as non-parametric limits of agreement between paired measurements. The significance level was adjusted using the Bonferroni correction (*p* value = 0.05/3 = 0.0167). For all other tests, a *p*-value of less than 0.05 was considered statistically significant. Sample size was calculated using the G*Power 3.0 program, with a confidence level of 95% and statistical power of 80%, using data from the study by Wongkanha et al. [29].

## 3. Results

A total of 136 pregnant women met the eligibility criteria and were included in the study.

### 3.1. Descriptive Data

The demographic characteristics of the participants are summarized in Table 1, including a mean maternal age of 28.32 ± 5.31 years, a median BMI of 26.33 kg/m^2^ (range: 18–40), a median gestational age of 140.5 days (range: 112–168), a median parity of 1 (range: 0–6), with 33.09% having a history of previous cesarean section, 30.15% being primigravidas, and 92.65% identified as of Turkish ethnicity.

### 3.2. Outcome Data

The comparison of UCA measurements obtained via three ultrasonographic approaches, TVUS, TPUS, and TAUS, is presented in Table 2. Repeated Measures ANOVA indicated a statistically significant difference among the methods (*p* < 0.001), with mean UCA values of 100.00 ± 24.02° for TVUS, 105.04 ± 25.70° for TPUS, and 91.99 ± 26.22° for TAUS. Post hoc analysis with Bonferroni correction revealed significant differences between TVUS and TPUS (*p* = 0.012), TVUS and TAUS (*p* < 0.001), and TPUS and TAUS (*p* < 0.001). The comparison of CL measurements across the three imaging techniques is presented in Table 3. Due to the non-normal distribution of CL values, the Friedman test was applied, revealing a statistically significant difference between methods (*p* < 0.001). The median CL was 35 mm (range: 20.6–55) for TVUS, 34 mm (range: 18.5–44) for TPUS, and 33 mm (range: 20–50) for TAUS. Bonferroni-adjusted Wilcoxon signed-rank tests demonstrated significant differences between TVUS and TPUS (*p* < 0.001) and between TVUS and TAUS (*p* < 0.001), while no significant difference was observed between TPUS and TAUS (*p* = 0.620) and the adjusted significance threshold set at *p* = 0.0167.

Pearson correlation analysis revealed significant positive correlations between UCA values obtained via TVUS and TPUS (r = 0.686; 95% CI: 0.585 to 0.765; *p* < 0.001), TVUS and TAUS (r = 0.547; 95% CI: 0.417 to 0.655; *p* < 0.001), and TPUS and TAUS (r = 0.464; 95% CI: 0.320 to 0.586; *p* < 0.001). Reliability analysis based on the ICC showed poor consistency between TVUS and TPUS (ICC = 0.052; 95% CI: −0.117 to 0.218; *p* = 0.273) and moderate consistency between TVUS and TAUS (ICC = 0.545; 95% CI: 0.415 to 0.653; *p* < 0.001).

**Figure 2:**
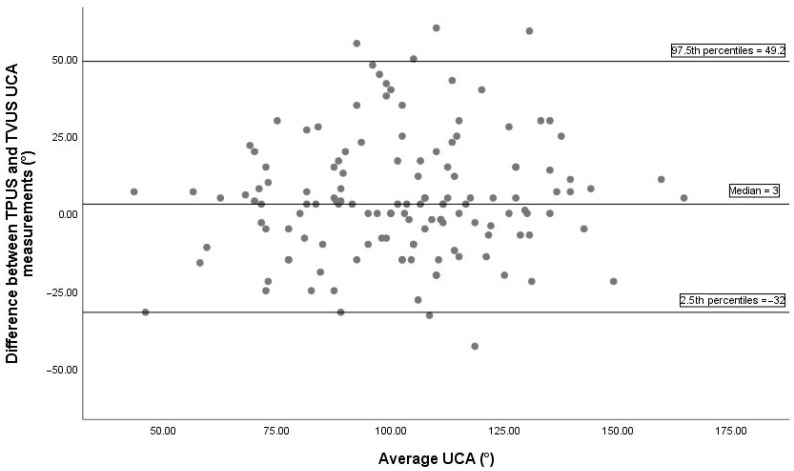
The Bland–Altman plot between TVUS and TPUS UCA measurements includes 136 pairs of successful measurements. The median difference between methods was 3°, indicating a minimal systematic bias. The 2.5th and 97.5th percentiles of the differences were −32° and 49.2°, respectively, representing the non-parametric limits of agreement. TPUS, transperineal ultrasonography; TVUS, transvaginal ultrasonography; UCA, uterocervical angle.

**Figure 3:**
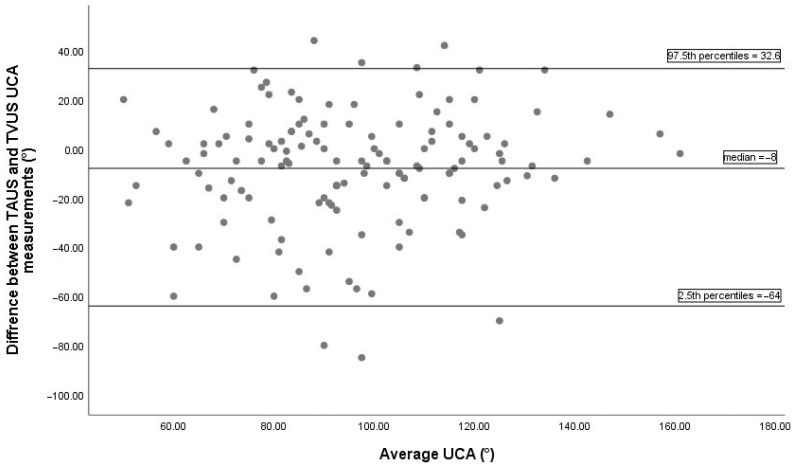
The Bland–Altman plot between TAUS and TVUS UCA measurements includes 136 pairs of successful measurements. The median difference between methods was −8°, indicating a minimal systematic bias. The 2.5th and 97.5th percentiles of the differences were −64° and 32.6°, respectively, representing the non-parametric limits of agreement. TAUS, transabdominal ultrasonography; TVUS, transvaginal ultrasonography; UCA, uterocervical angle.

Bland–Altman analysis comparing UCA measurements between TVUS and TPUS, based on 136 paired observations, is illustrated in Figure 2. The median difference between the two methods was 3°, indicating minimal systematic bias. The 2.5th and 97.5th percentiles of the differences were -32° and 49.2°, respectively, indicating the non-parametric limits of agreement. The Bland–Altman plot comparing UCA measurements between TVUS and TAUS is displayed in Figure 3. The median difference was −8°, showing a slight systematic bias, with non-parametric limits of agreement ranging from −64° to 32.6°.

## 4. Discussion

### 4.1. Principal Findings

Our study demonstrated that UCA measurements vary significantly depending on the ultrasound method used. Although strong positive correlations were observed between UCA values obtained via different modalities, reliability analysis revealed only moderate consistency between TVUS and TAUS (ICC = 0.545) and poor consistency between TVUS and TPUS (ICC = 0.052). These findings suggest that while all three methods can similarly track UCA changes, TPUS introduces considerable variability compared to the gold standard, TVUS.

### 4.2. Interpretation

This study presents the first evaluation of UCA measurement using all three ultrasound modalities (TVUS, TAUS, and TPUS). A similar study by Wongkangha et al. [29] assessed the correlation between TVUS and TAUS but did not include TPUS. Both studies confirmed significant correlations between UCA values obtained through TVUS and TAUS. Wongkangha et al. reported Pearson correlation coefficients of 0.438 (prevoid) and 0.601 (postvoid) between TAUS and TVUS, while our study demonstrated a moderately stronger correlation of 0.547. This similarity underscores the potential of TAUS as a surrogate for TVUS in clinical practice. However, a key methodological distinction lies in bladder status during measurement: Wongkangha’s study compared pre- and postvoid bladder states, revealing that a full bladder increases the UCA measurement, while in our study, all measurements were performed with an empty bladder, and the effect of bladder filling was not evaluated. One of the key differences between the two studies is the statistical methodology employed. While Wongkangha’s study relied solely on correlation analysis, our study additionally utilized ICC and Bland–Altman analyses to assess measurement agreement and consistency [29]. High correlation alone does not demonstrate that two methods are consistent or interchangeable. Reliability analysis with ICC should be used to assess consistency, and agreement should be evaluated with a Bland–Altman plot. Correlation may be high while ICC is low. A systematic bias may result in a high correlation but a low ICC. In our study, ICC analysis revealed moderate consistency between TAUS and TVUS (ICC = 0.545; 95% CI: 0.415–0.653; *p* < 0.001). This result suggests that TAUS not only shows correlation but also provides a certain level of reliability in terms of reproducible measurements. According to the Bland–Altman analysis, the median difference in UCA measurements between TAUS and TVUS was −8°, indicating a systematic negative bias; meaning that TAUS tends to measure lower angles compared to TVUS. Although this bias falls within statistically acceptable limits and suggests a reasonable level of agreement between the two methods, the wide limits of agreement (approximately −64° to +32.6°) point to considerable variability at the individual level. This implies that while TAUS may be suitable for population-level research where average values are compared, it should be used cautiously in individual clinical decision-making due to the potential for significant discrepancies in single measurements.

In our study, although UCA values obtained via TPUS showed a significant correlation with TVUS (r = 0.686), reliability analysis revealed poor consistency (ICC = 0.052). This suggests that while TPUS may reflect general trends in UCA changes, it lacks the precision necessary for standalone clinical use. The findings of the Bland–Altman plot also support this observation. The Bland–Altman plot comparing TPUS and TVUS revealed a wide range of differences (−32° to +49.2°), indicating substantial inter-individual variability in measurements. This broad limit of agreement demonstrates limited agreement between the methods, suggesting that they cannot be used interchangeably for individual patient assessments.

There is no other study evaluating the correlation between TPUS and TVUS for UCA measurement; however, Songserm et al. compared CL measurements using TPUS and TVUS in pregnancies between 16 and 24 weeks of gestation. In the study conducted by Songserm et al., a high correlation (r = 0.94) and strong agreement (Lin’s CCC = 0.94, interobserver ICC = 0.98) were reported between CL measurements obtained by TVUS and TPUS methods [27]. These findings suggest that TPUS can be a reliable alternative for CL assessment. However, in our study, although a moderate correlation (r = 0.686) was found between TPUS and TVUS for UCA measurements, the ICC value was quite low (ICC = 0.052), indicating a lack of significant consistency between the methods. There may be several reasons for the differences in results between the studies. While CL is measured as a direct linear distance, UCA is an angular measurement and therefore more sensitive to anatomical variations and measurement technique. Especially in externally applied methods like TPUS, movement of perineal soft tissues, probe positioning, and pressure can affect angular measurements and lead to inconsistency. Although both studies minimized interobserver variability by using a single experienced operator, achieving intraobserver agreement in angular measurements can be more challenging. Consequently, while TPUS is accepted as a reliable and close alternative to TVUS for CL measurement, it shows limitations in assessing angular and more complex structural parameters such as UCA due to technical and anatomical factors. These differences highlight the need for standardization and technical improvements in TPUS for UCA measurement. Further studies optimizing these methods and validating them in larger patient populations will enable more reliable use of UCA in obstetric practice.

TVUS has been the primary method for UCA measurement in both clinical practice and research, similar to its role in CL assessment. According to our findings, TAUS showed moderate agreement with TVUS and may be considered a practical and more acceptable alternative for patients, particularly when TVUS cannot be performed. In contrast, TPUS demonstrated high variability and is not suitable for routine clinical use; it may only be considered after further studies achieve standardization. Mechanistically, the variability between the methods arises from anatomical and technical factors. TVUS provides the closest and most direct visualization of the cervix, minimizing the effects of maternal tissues or fetal position. TAUS, while non-invasive and accessible, can be influenced by factors such as bladder fullness, fetal position, and uterine angle. In cephalic presentation, the fetal head exerts pressure on the cervix and lower uterine segment, whereas in breech presentation, the pressure pattern and imaging shadows differ. This can affect the clarity and reliability of measurements performed with TAUS. TPUS presents a more complex measurement environment due to labial/perineal tissues and probe pressure, which reduce its reliability. One of the main technical limitations of TPUS is the insonation angle. When the cervix is aligned with the vaginal axis and the ultrasound beam is perpendicular to the external os, accurate UCA measurement becomes challenging. Furthermore, air in the vaginal canal can cause scattering at tissue interfaces, leading to artifacts. We recommend that future studies be conducted in a multicenter setting with an assessment of interobserver variability. Furthermore, we suggest comparing the three ultrasonographic methods in pregnancies beyond 24 weeks and investigating the impact of UCA measured by TPUS and TAUS on the prediction of spontaneous preterm birth. Additionally, we suggest conducting studies on the clinical implications and measurement of the angulation between the cervix and the vagina.

### 4.3. Strengths and Limitations

Key strengths of our study include its prospective design, the consecutive application of three ultrasound methods in a standardized setting, and a robust statistical methodology. Additionally, being the first study to compare all three methods for UCA measurement and to evaluate TPUS contributes significantly to the literature. Measurements performed by a single experienced operator minimized interobserver variability and enhanced measurement consistency. Some limitations of our study should be noted. The measurements were performed by a single obstetrician; this means that interobserver variability was not assessed, which may limit the generalizability of the study. Additionally, having the same operator conduct all measurements and be aware of previous results could have influenced subsequent measurements, potentially introducing bias. Since our study included a low-risk patient group for spontaneous preterm birth, it does not provide data to make inferences about high-risk patients. Our findings are limited to pregnancies between 16 and 24 weeks. The predictive value of UCA measurements for spontaneous preterm birth using TPUS and TAUS is unknown. Furthermore, conducting our study at a single center represents another limitation regarding generalizability.

## 5. Conclusions

Our study demonstrated that TAUS showed a moderate correlation and moderate consistency with the gold-standard TVUS in measuring UCA. In contrast, TPUS exhibited substantial variability and poor agreement with TVUS. Our findings need to be validated by comprehensive studies. Additionally, there is a need for studies that separately evaluate the predictive value of UCA measurements obtained using TPUS and TAUS, apart from TVUS, for spontaneous preterm birth.

## Figures and Tables

**Table 1:** Demographic data.

Demographic Data	n: 136
Maternal age ^a^ (year)	28.32 ± 5.31
BMI ^b^ (kg/m^2^)	26.33 (18–40)
Gestational age ^b^ (days)	140.5 (112–168)
Parity ^b^	1 (0–6)
Previous cesarean section ^c^ n	45 (33.09)
Primigravidas ^c^ n	41 (30.15)
Turkish ethnicity ^c^ n	126 (92.65)

Abbreviations: BMI, body mass index. ^a^ Normal distribution, Mean ± SD; ^b^ Non-normal distribution, Median (Minimum–Maximum); ^c^ Categorical data, Number (Percentage).

**Table 2:** Comparison of different ultrasonographic methods in UCA measurement.

ANOVA (Repeated Measures) Test (n: 136)			Bonferroni Post Hoc Test	
	Mean ± SD	*p* Value	Pairwise Comparison	*p* Value
TVUS UCA (°)	100 ± 24.02	<0.001	TVUS-TPUS UCA (°)	0.012
TPUS UCA (°)	105.04 ± 25.70		TVUS-TAUS UCA (°)	<0.001
TAUS UCA (°)	91.99 ± 26.22		TPUS-TAUS UCA (°)	<0.001

Abbreviations: TAUS, transabdominal ultrasound; TPUS, transperineal ultrasound; TVUS, transvaginal ultrasound; UCA, uterocervical angle.

**Table 3:** Comparison of different ultrasonographic methods in CL measurement.

Friedman Test (n: 136)			Bonferroni-Corrected Wilcoxon Test	
	Median (min-max)	*p* Value	Pairwise Comparison	*p* Value
TVUS CL (mm)	35 (20.6–55)	<0.001	TVUS-TPUS CL (mm)	<0.001
TPUS CL (mm)	34 (18.5–44)		TVUS-TAUS CL (mm)	<0.001
TAUS CL (mm)	33 (20–50)		TPUS-TAUS CL (mm)	0.620

Abbreviations: CL, cervical length; TAUS, transabdominal ultrasound; TPUS, transperineal ultrasound; TVUS, transvaginal ultrasound. Significance level adjusted using Bonferroni correction (*p* value = 0.05/3 = 0.0167).

## Data Availability

The data presented in this study are available on request from the corresponding author due to privacy and ethical restrictions.

## Abbreviations

AUC	Area under the curve
BMI	Body mass index
CL	Cervical length
ICC	Intraclass correlation coefficient
ISUOG	International Society of Ultrasound in Obstetrics and Gynecology
TAUS	Transabdominal ultrasound
TPUS	Transperineal ultrasound
TVUS	Transvaginal ultrasound
UCA	Uterocervical angle
